# LARNet: Real-Time Detection of Facial Micro Expression Using Lossless Attention Residual Network

**DOI:** 10.3390/s21041098

**Published:** 2021-02-05

**Authors:** Mohammad Farukh Hashmi, B. Kiran Kumar Ashish, Vivek Sharma, Avinash G. Keskar, Neeraj Dhanraj Bokde, Jin Hee Yoon, Zong Woo Geem

**Affiliations:** 1 Department of Electronics and Communication Engineering, National Institute of Technology, Warangal 506004, India; mdfarukh@nitw.ac.in; 2 Viume, Hyderabad, India; krnkumar663@gmail.com; 3 Indian Institute of Information Technology, Nagpur 441108, India; sharmavivek7866@gmail.com; 4 Department of Electronics and Communication Engineering, Visvesvaraya National Institute of Technology, Nagpur 440010, India; agkeskar@ece.vnit.ac.in; 5 Department of Engineering—Renewable Energy and Thermodynamics, Aarhus University, 8000 Aarhus, Denmark; 6 Department of Mathematics and Statistics, Sejong University, Seoul 05006, Korea; jin9135@sejong.ac.kr; 7 Department of Energy IT, Gachon University, Seongnam 13120, Korea

**Keywords:** facial micro expressions, LARNet, microscaling level, feature extraction, lossless attention network

## Abstract

Facial micro expressions are brief, spontaneous, and crucial emotions deep inside the mind, reflecting the actual thoughts for that moment. Humans can cover their emotions on a large scale, but their actual intentions and emotions can be extracted at a micro-level. Micro expressions are organic when compared with macro expressions, posing a challenge to both humans, as well as machines, to identify. In recent years, detection of facial expressions are widely used in commercial complexes, hotels, restaurants, psychology, security, offices, and education institutes. The aim and motivation of this paper are to provide an end-to-end architecture that accurately detects the actual expressions at the micro-scale features. However, the main research is to provide an analysis of the specific parts that are crucial for detecting the micro expressions from a face. Many states of the art approaches have been trained on the micro facial expressions and compared with our proposed Lossless Attention Residual Network (LARNet) approach. However, the main research on this is to provide analysis on the specific parts that are crucial for detecting the micro expressions from a face. Many CNN-based approaches extracts the features at local level which digs much deeper into the face pixels. However, the spatial and temporal information extracted from the face is encoded in LARNet for a feature fusion extraction on specific crucial locations, such as nose, cheeks, mouth, and eyes regions. LARNet outperforms the state-of-the-art methods with a slight margin by accurately detecting facial micro expressions in real-time. Lastly, the proposed LARNet becomes accurate and better by training with more annotated data.

## 1. Introduction

**Figure 1:**
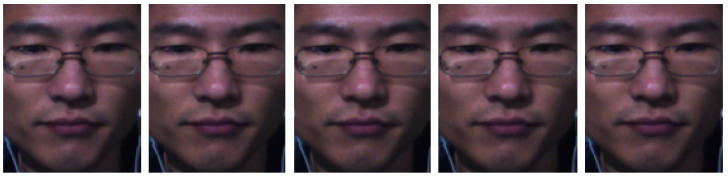
Sample micro expression from SMIC dataset [3].

Facial expressions are one of the most important aspects of human communication, especially in commercial spaces [1]. These expressions contribute to communicating and understanding not only the emotional take of a person but also the person’s actual ideas and thoughts, which he/she may not be willing to share. A crucial feature of facial expressions or facial emotions that makes this study so valuable that they are almost the same universally, irrespective of geography. Facial macro expressions are easily identified by humans and easily displayed. This fact results in questioning the genuineness of emotions, as those are easy to generate and hence can be used in deception. This is where micro expressions come into the picture. According to psychologists and researchers, micro expressions are facial expressions that show the true emotions of a person. Those facial expressions can also be termed as emotional leakage. Micro expressions are spontaneous and reveal a person’s true emotion in that context. This display of true emotions through micro expressions surfaces for a brief time only, for 1/15th to 1/25th of a second. This is so quick and spontaneous that it can hardly be noticed with the naked eye. The main challenge [2] is that micro expressions are complex to identify with the naked eye, yet no one can hide them. Figure 1 shows a sample of micro expressions of negative feelings. A micro expression classification would classify the sample as normal, but deep inside, the real emotion is that of a negative feeling. Hence, an in-depth analysis is needed for real feedback from customers maintaining a smooth commercial operation with a profit margin. As there is a tremendous increase in retail space, actual customer feedback on any product is a deciding factor for a product to be manufactured with the attached features. Although commercial complexes have deployed facial recognition and implemented facial expression recognitions, often, facial macro expressions do not indicate true emotions. Facial micro expressions are organic, i.e., spontaneous and will be upheld only for a very minute fraction of a second. However, they display the actual emotions and are crucial for feedback on products or situations. Hence, facial micro expressions are widely promoted for research and commercial usages. This model is even tested with a lie detector using a vision-based approach, as micro expressions properties can properly define actual emotion. Hence, a vision era on detecting real emotions extracted from micro-scale features are in growing demand in commercial, research, and defense fields.

Many image classification architectures have been developed in the recent past and proven to provide a satisfactory result on macro expressions. However, they fail to work when accurately identifying facial micro expressions, as micro expressions are held just for a micro fraction of a second and need a depth micro-scale feature extraction for training. This work summarizes the depth in which residual attention networks perform on micro expressions and how they extract micro-scale features from a dataset.

## 2. Background and Related Work

Due to its genuineness and diversified use, research on micro expression have gained momentum in the recent years. The field of computer vision and pattern recognition has attracted many researchers to work on this topic due to its sparse usage in the commercial and psychological spaces.

The pattern recognition of the micro expressions has been mainly analyzed based on major six emotions. Micro Expression testing was first done on the database presented by Polikovsky [4], York Deception Test [5], and USF-HD [6]. But these datasets being insufficient were soon overtaken by SMIC [3], CASME II [7], CASME [7], and CAS(ME)2 [8]. The main reason the former did not gain popularity because the datasets were created by asking the participants to mimic or create emotions which as explained before does not generate micro expression. These were mainly artificial type of emotions and not the real ones. Hence, no fruitful results can be concluded using the former datasets. The York DDT contained very few expressions which were clearly insufficient for the research. The dataset SAMM, which stands for spontaneous actions and micro movements, consisted of 32 participants from nearly 13 different cultures. These datasets, rather than focusing on emotion recognition, focused on micro movement identification.

### 2.1. Traditional Approaches

The feature extraction technique evolved over the years due to easily available dataset and ever going research in face forensics field. Among these techniques is the LBP (Local Binary Pattern) introduced by Ojala et al. [9]. LBP produced a remarkable result on monotonic illumination variation but limited to spatial data. So, to gain results in low intensity value Local Binary Patterns on Three orthogonal Planes (LBP-TOP) was introduced. LBP-TOP is basically the upgraded form of the first introduced LBP which now works on both temporal and spatial feature extraction simultaneously. Li et al. [10], Yan et al. [7], Pfister et al. [11], Guo et al. [12], House and Meyer [13], and Adegun and Vadapalli [14] implemented LBP-TOP features extraction with different facial detection and classification method for micro expression detection. The main drawback of TOP model was the computational complexity and hence efforts to improve the performance led to development of LBP-SIP or Linear Binary Pattern with Six Intersection Points and LBP-MOP (with Mean Orthogonal Planes). The drawbacks of these methods are the accuracy in extracting micro-scale level features on facial micro expressions due to limited scale features being extracted and trained.

### 2.2. Deep Learning Approaches

Deep learning-based approaches have gained attention in face forensics recently, particularly in the detection fields. A high-level representation of micro expressions can be extracted from Convolution Neural Network (CNN)-based algorithms. Patel et al. [15] were the first to introduce a CNN model in facial micro expressions detection. Due to fewer usable datasets, the researchers used pre-trained ImageNet weights with the Visual Geometry Group (VGG) architecture model. Mayya et al. [16] introduced another method in their proposed model by combining temporal interpolation with a deep CNN (DCNN) for recognition. Later, it was fed to support vector machine (SVM) for classification and for faster performance using a Caffe [17] library, which was used for feature extraction along with a Graphics Processing Unit (GPU) unit. The advantages of image classification using transfer learning containing feedforward convolution networks are using very deep structures [15,18,19] and decoder functionality in auto encoder which is later taken from the feedforward mechanism. Further, several methods have been proposed for improving the discriminative ability of deep convolutions, such as VGG [15], Inception [19], and residual learning [18]. To avoid overfitting and to exploit regularization for convergence, functions, such as stochastic depth [20], batch normalization [21], and dropout [22], have been initialized. However, all of the above models could not capture critical micro-scale movements in micro expressions datasets.

Hence, deep learning-based approaches have gained potential in the face forensics in the recent past. The first framework in the field of face recognition was introduced by Jones-Viola [23]. Their framework detected faces in an image using machine learning approach in real time. After that a large number of CNN-based face detection methods have been developed including Normalized Pixel Difference (NDP) face [5]. Among them was one proposed by Ranjan et al. [24] which used a selective search algorithm for face detection. It was although not able to localize well with the actual face region. The deep learning mechanisms have gained lot of attraction in various detection fields. Facial recognition and micro expression field is not less in this. The high-level representation of micro expressions is extracted from convolution neural networks-based algorithms. Patel et al. [15] were the first to introduce CNN model in facial micro expressions detection. Due to less usable datasets, the researchers used pre-trained ImageNet weights with VGG architecture model. Mayya et al. [16], in their proposed model, introduced another method by combining temporal interpolation with deep convolutional neural network (DCNN) for recognition. Later, it was fed to SVM for classification for a faster performance using Caffe [17] library which was used for feature extraction along with GPU unit. Recent advantages on image classification using transfer learning containing feedforward convolutions networks are using very deep structures [15,18,19] and the decoder functionality in auto encoder which is later taken from the feedforward mechanism. Several methods have further been proposed to improve the discriminative ability of deep convolutions, such as VGG [15], Inception [19], and residual learning [18]. To avoid overfitting, functions, like stochastic depth [20], batch normalization [21], and dropout [22], have been initialized and to exploit regularization for convergence. However, all the above models could not capture the critical micro-scale movements of micro expression datasets.

In recent times, region proposal networks [25,26,27,28,29] has been successfully adopted in object detection applications. In image classification, an additional region proposal stage [30] is added before feedforward mechanism. The proposed regions contain useful information and are hence used for feature learning in the further stages. Unlike object detection, in which its region proposals rely the ground truth bounding boxes or detailed segmentation masks [31], unsupervised learning [32] is usually used to generate region proposals for image classification. But, due to the heavy complexity of bringing-in segmentation masks and boundary boxes, especially for image classification tasks, this model is completely unnecessary.

Peng et al. [33] proposed a model called dual temporal scale CNN for recognizing spontaneous micro expressions. This network works in two streams. These streams are used to process multiple frame rates of a micro expressions dataset. Each stream contains an independent shallow network to estimate overfitting. Inputs can be optical flow sequences, so that features can be produced by a shallow network. After learning, a linear SVM feature classifier is used to classify the output. The model has been proven to show decent performance compared with the conventional naive SVM and LBP methods, but it experiences the same problem with lagging in the extraction of critical micro-scale features in the model because of which its accuracies is not high enough to proceed.

Kim et al. [34] proposed a model consisting of CNN and long short-term memory (LSTM) to manage spatial and temporal information. Instead of using full movement intensity, each expression stage is learned by the network in the spatial domain. The variation in expression classes, state, and state continuity results in making features resistant to variation in illumination. LSTM helps in learning the CNN spatial information and its temporal characteristics. The LSTM approach can extract temporal information through distinct frame rate video datasets. The developed model obtained better accuracy than the old LBP techniques and subsequent variant models. Although, the imbalance in the dataset samples affected the confusion matrix results. Control gates have been used extensively in LSTM networks. In the process of feedforward training, updates are made in control gates for neurons using the helpful information. Further, the control gates have a direct influence in this process [25,26]. Choi et al. [35] proposed LFM-based CNN-LSTM hybrid method to recognize facial micro expressions from video frames. Landmark feature maps (LFM) extracts landmarks from all parts of the face and is then fed to the CNN-LSTM hybrid architecture to compute and classify the facial micro-expressions. Although the architecture is computationally strong enough to dig deeper into the frames, the major drawback is it equally focus on all parts instead of the parts which change with respect to emotions frame-wise.

Recently, Yu et al. [36] introduced a deep cascaded peak pilot network to learn and determine weak expressions. Apex, i.e., peak expressions were used to supervise onset/offset non-peak expressions. The addition of backpropagation and a cascaded fine-tuned algorithm improved the overfitting problem and performance simultaneously. However, the authors tested macro expressions, which resulted in a best performance of approximately 90%.

Soft attention networks [37,38] developed in recent times [39,40] and soft attention modules are employing residual attention networks to develop a feedforward neural network. This approach has been adopted by the authors for this work. Recently proposed spatial transformer modules by Jaderberg et al. [40] achieved contemporary results on almost all visual recognition tasks. An affine transformation is produced by a residual network that captures useful information available in the encoder section. Then, the input image patch is processed with the affine transformation to determine the attended region. Further, it is fed to the residual network for feature extraction.

This process is performed in an end-to-end residual attention framework that performs spatial transformations. This work has been inspired by Wang et al. [41] regarding the design of soft attention networks with encoders and decoders as the pipeline for extracting top feature maps from both global and local information. Long et al. [42] performed skip connections, which were used within the top and bottom features and reached state-of-the-art image segmentation results. Although this approach works satisfactorily, image classification does not require high weight structures that consume high computation power. Hence, much into local information as image segmentation, this work focuses on global and local information as far as micro-scale features from the face are included. The dataset consists of several videos, and each video is only a few seconds long, i.e., when a specific expression is seen, a video is recorded. This temporal information is considered for model training. Hence, the dataset is well refined, as micro expressions cannot be easily identified by cropping a video to the particular segment which contains the expression.

## 3. Technical Approach

Facial micro expressions detection using Lossless Attention Residual Network (LARNet) is an end-to-end deep learning framework for classifying underlying facial microemotions. These expressions might not be captured by a human owing to their instantaneous change. Hence, the proposed model is fed with consecutive frames of the video, whereby each frame shows a very minute fraction of change. This change is the key to extracting information from the frames. LARNet extracts this crucial information, which is available for a fraction of a second, and trains it accordingly under a specific class label. This is even applicable to detecting unconscious emotions.

LARNet is constructed as a stack of multiple attention modules similar to the residual attention networks mentioned in Reference [41], whereby each branch is classified into two sub-branches, named as mask and trunk branches. Feature extraction processing is performed in the trunk branch and this block is adapted by comparison with other state of the art feature extraction processing methods. In this work, the authors have implemented two residual blocks, ResNet-56 and ResNet-92, concatenated with a custom-designed residual block built on ResNet, known as EmoResNet. The two residual networks were used as already built, and the authors froze their last layers and concatenated them with the upcoming layers, in this case, the next blocks. The outputs of each residual block are fed as inputs to the other, and the latter is fed to EmoResNet. Input *x* is given to the trunk branch which produces an output T(x). The mask branch computes a generation of masks on each image using a bottom-up and top-down approach, which mimics the feedforward and feedback attention process. Control gates of neurons in the trunk branch are the result of the outputs of the mask branch, i.e., mask outputs are bridges with control gates similar to a highway network [41]. Attention network outputs are represented as:

(1) $$H_{i,c}\left(x\right)=M_{i,c}\left(x\right)*T_{i,c}\left(x\right),$$

where *i* varies according to the overall spatial positions, and the index of the channel is defined as c∈{1,2,⋯,C}. *H* represents the output of the attention module, *M* displays the mask size in the mask branch, and *T* is the trunk output branch.

The backbone of the mask branch serves as the feature selector during the feedforward mechanism and as a gradient update filter during the backpropagation process. The gradient for input feature selection in the mask branch is defined as

(2) $$\frac{\partial M(x,\theta )T(x,\phi )}{\partial \phi }=M\left(x,\theta \right)\frac{\partial T(x,\phi )}{\partial \phi },$$

where θ and ϕ are the mask and trunk branch parameters, respectively. The trunk branch parameter mainly consists of a convolution filter. The advantages of having a mask branch in the attention network are that the wrong gradients are prevented from the dataset and trunk parameters are updated if noisy labels are present in the dataset [41]. The mask branch uses up-sampling and down-sampling computation to prevent any wrong gradients. The top-bottom approach can then identify wrong gradients and update the trunk branch accordingly. The authors have created a soft weight mask by implementing a three-network residual branch that is identical to the layer of spatial transformer. As the network clusters the features from the face, the drawbacks faced with existing state-of-the-art models, such as cluster background, complex scenes, zoomed appearance, etc., would require considerable attention, thereby making the network more complex. The main drawback of existing attention models that they can modify features only once using the backpropagation channel. The network does not have a scope for further modification if it fails either in some part or entire image. This results in false features and inaccurate results.

Hence, the authors have introduced three residual blocks that alleviate the single check feature extraction. Each trunk branch in the attention model uses its mask branch for feature learning.

### 3.1. Attention Residual Learning

This section describes the feature learning methodology of attention modules. There is an apparent performance drop in naive attention networks. This apparent drop takes place owing due to the degradation of matrix values of features in the hidden layers caused by a repeated dot product of the mask range from zero to one. There is a conception that masks branch breaks identical mapping of a residual unit of the trunk branch in naive attention modules.

These problems can be eradicated if the output from the attention network can be modified as follows.

(3) $$H_{i,c}\left(x\right)=\left(1+M_{i,c}\left(x\right)\right)*F_{i,c}\left(x\right).$$

Here, M(x) varies from [0, 1], such that M(x) and H(x) approximating 0 and features F(x), respectively. This is a representation of residual learning.

The original concept of residual learning proposed through ResNet was formulated as $H_{i,c}\left(x\right)=x+F_{i,c}\left(x\right)$, designating $F_{i,c}\left(x\right)$ as the residual function. This proposal slightly tweaks the function in mapping the features generated by the ConvNets, inspired by Wang et al. [41]. This implicates the mask branch being identical in terms of feature mapping and selectors to increase good features and removes the noise from extracted features with the trunk branch. Unlike a single run feature modification, stacking attention network backs up in tweaking its weights in an incremental manner. This network extracts good properties from extracted valid features, bypasses the soft mask branch, and then weakens the mask branch’s feature extractor. This gives the network the ability to go deeper into the features, thereby consistently increasing accuracy. A similar type of work implemented by Wang et al. [41] surpassed the performance of other residual networks by 452 times.

### 3.2. Mask Branch Block

Moving forward with the idea of an attention mechanism as proposed by Larochelle et al. [43], the authors have instigated fast feedforward and top-down feedback steps for extracting good features and valid weights to attain a near-zero error rate. As mentioned in the previous section, the mask branch plays an important role in the feature extraction process. The feedforward block accumulates global information from the image and the top-down feedback block combines this global information with the feature maps. The max-pooling function is used in the input block in all small residual modules to increase the receptive field swiftly. When the images reach the lowest resolution while feature extraction is similar to an encoder network, the global information is drastically expanded symmetrically by the top-down feedback block to direct the input features at each pixel block level. The sigmoid activation function is then attached at the branch end, and it normalizes the output to the range of [0, 1] coming from two consecutive 1×1 Conv layers. Skip connections are added between fast feedforward or top-down and bottom-up layers to capture information through features from different scales. Top-down and bottom-up networks in the residual attention module gear the entire network to learn features better for micro-scale level feature learning through branch blocks.

### 3.3. Spatial and Channel Attention

Three types of activation functions are used in this architecture: mixed, channel, and spatial attention. These comprise the module because the mask branch updates abruptly with the features of trunk branch. To normalize this, the above-mentioned activation functions are used before the mask branch outputs. Let m(f) represent a mixed attention function, c(f) as the channel attention function, and s(f) as the spatial attention function. m(f) uses the sigmoid activation function in each channel with a spatial position. The spatial information is removed as c(f) performs L2 normalization in all channels. Normalization is performed by s(f) on feature maps in each channel, and then passes it the sigmoid activation function to obtain spatial information from the soft mask blocks [41].

Table 1 shows the experimental results of all three attention activation functions used on the CASME2 micro expressions dataset. Due to the unavailability of a large-scale dataset of facial micro expressions, a limited scale dataset has been used for experimental trials. Many previous works implemented the latter two activation functions in their proposed residual attention networks, which resulted in stroking complex constraints on weights in the soft mask branch. This can be eradicated, as implemented in this work, by adaptively changing the attention modules with the extracted features, which provide the best performance. Equations (4)–(6) are experimenting with activation functions present in the soft mask branch. Equation (5) represents the channel attention that exploits the inter-channel relationship of the features. It mainly focuses on detecting useful information from the data and squeezes the spatial dimension of the input feature maps. Equation (6) represents the spatial attention that uses the inter-spatial relationships of the features. It mainly focuses on the location of useful information from the data. Equation (4) is a mixed attention function, which mimics both Equations (5) and (6), and is a hybrid mechanism of the channel and spatial functions for reducing the error rate. Useful information is detected and extracted with low error rates using this hybrid mechanism. Hence, the mixed function outperforms all others and is performed sideways with the convolution functions.

(4) $$m\left(f\right)\left(x_{i,c}\right)=\frac{1}{1+exp(-x_{i,c})},$$

(5) $$c\left(f\right)\left(x_{i,c}\right)=\frac{x_{i,c}}{∥x_{i}∥},$$

(6) $$s\left(f\right)\left(x_{i,c}\right)=\frac{1}{1+exp(-\left(x_{i,c}-mean_{c}\right)/std_{c})}.$$

Here, *c* ranges over all channels, and *i* varies according to all spatial positions. The mean and standard deviation for the feature map from the *c*th channel are denoted by $mean_{c}$ and $std_{c}$, respectively. $x_{i}$ denotes the feature vector and the *i*th spatial position.

In stage 1, convolution processing of the given input data is intensively computed with the ImageNet pre-trained weights. This stage initially computes the information and stores it in a feature vector. As facial micro expressions require intense extraction of features, and not all feature vectors are useful for detecting minute micro expressions, the feature vectors are further fed to stage 2, in which soft mask and trunk branches are present with a mixed attention activation function. This stage is useful for locating and extracting information from the feature vectors. The curated feature vector is finally computed with convolution filters in stage 3.

## 4. Experiments and Results

### 4.1. Network Construction

The proposed three-module network was used for the performance evaluation of the proposed LARNet on a series of benchmark datasets, including CASME II [7,8], USF-HD [44], and SMIC [3].

**Figure 2:**
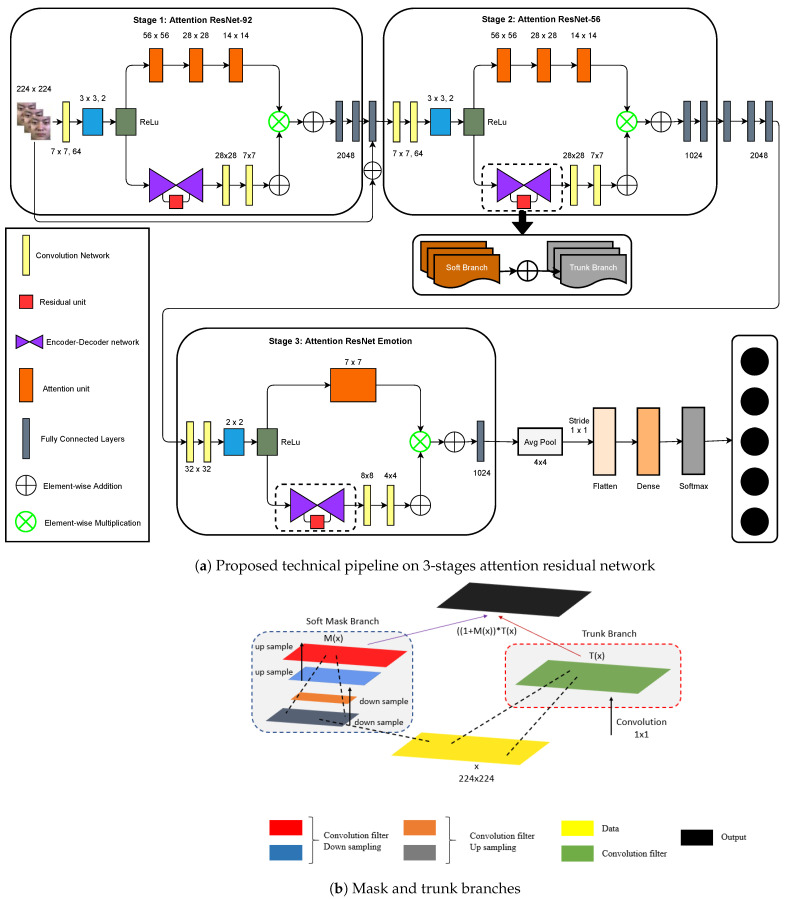
Proposed technical pipeline.

The model is constructed by stacking all three attention modules as shown in Figure 2a, starting with ResNet-92, ResNet-56, and followed by the customized Attention EmoResNet. The former two blocks take the input shape 224 × 224 × 3, with the number of image channels being 64 with a dropout = 0 and regularization = 0.01; for the two modules in their first layer. Both modules are fed and computed with the L2 regularization penalty.

The L2 regularization penalty is computed as:

loss=L2×reduce_sum(square(x)).

Figure 2b represents the inside architectural view of the mask and trunk branches. The mask branch has up sampling and down sampling connections from the inputs, and the trunk branch resides with the convolutional functionality. Convolutional filters in the soft mask branch compute downsampling and upsampling layers with the max-pooling layer between each of them. The data are further processed to extract deeper information through up-down sampling. The trunk branch contains a single layer of the convolutional layer. The output of the two branches’ are concatenated and further sent to stage 3, that is, EmoResNet. The main use of the soft mask branch is to reduce the error rate and benefit from multi-scale information. The trunk branch performs local convolutions.

#### 4.1.1. Attention ResNet-92

In the ResNet-92 attention module, the cropped image size of 224 × 224 is fed to a 2D-Conv channel parametrized in a 7 × 7 kernel size, 64 image channels, a stride of size 2 × 2 with padding of 1. The output matrix was of size 112 × 112; a total of 5000 datasets are given as training datasets. Each time, the total number of input images are fed to the network with respect to the batch size, which is 64, with the initial image channel as 3 with an input image size of 224 × 224. This is further fed for computation with a 2D-Conv channel parametrized with 7 × 7 kernel size, 64 image channels, stride of size 2 × 2 with padding = ‘1’. Resulting in an output matrix size of 112 × 112 with output channels as 64, batch normalization was applied to re-scale and re-center along with a ReLu activation function, and a 2-D max-pooling, parameterized with a pool size of 3 × 3, stride = 2 × 2 and padding = ‘1’, emitting the first layer output in a 56 × 56 matrix size. This was then passed to the second layer containing a residual block, with four times as many output channels as the initial image channels, that is, 4 × 64 (previous output channels size) = 256. Regarding the output size, the residual block = 56 × 56, this is passed as input to the attention block with encoder depth = 3, resulting in a bottleneck of size 7 × 7 as a final second-layer output. This is further given to the third layer consisting of one residual block and two bottleneck attention blocks. The third-layer residual block comprises eight times the initial image channels, that is, 512, with stride = 2 × 2, resulting in an output size of 28 × 28. This is passed to two attention blocks, each with an encoder depth of 2, resulting in a bottleneck of size 7 × 7, thus resulting in total output size from the third layer of 7 × 7. The fourth layer consists of one residual block and three attention blocks. The residual block has 16 times the initial image channels, namely 1024, with stride size 2×2, resulting in a 14×14 output. This is fed to the attention block. There are three attention blocks, each with an encoder depth of 1, resulting in a bottleneck of size 7×7, thus resulting in a final output size of 7×7. The final residual block layer implies three residual blocks, each with 32 times as many output channels as the initial image channel, that is, 2048, with the first block with stride size =2×2, resulting in an output size of 7×7. The final layer of this module holds a pool size, comprehending the first and second indices of the present result, by consuming average 2-D pooling with the mentioned pool size and stride =1×1, and then the flattened function and dropout activation, fleeting it to the output node dense with 7 output nodes (for 7 classes), kernel regularizer, and softmax activation function.

#### 4.1.2. Attention ResNet-56

Using the ResNet-56 architecture as a backbone, as mentioned above, that takes input image size is 224×224, with initially 64 image channels, a dropout = 0, and an L2 regularization penalty. Starting with the first layer, Conv 2D with 64 image channels, a 7×7 kernel size with stride =2×2 and padding = 1, results in an output size of 112×112. This is then given to the batch normalization function aligned with the ReLu activation function. Further, it is passed on to maximum 2-D pooling with a pool size of 3×3, stride of 2×2, and padding = 1, resulting in an output size of 56×56. The second layer opening with the residual blocks has one residual block and an attention block. The residual block has a total of four times the output channels of 4 the initial image channels, that is, 256, resulting in the total output size decreasing to 56×56. This is fed to the attention block with an encoder depth of 3, reaching a bottleneck of size 7×7. The third layer starts with the residual block with eight times the number of output channels as the initial image channels, summing to 512, with a stride =2 and resultant matrix size of the residual block of 28×28. The attention block, in contrast, has an encoder depth of 2, reaching a bottleneck of size 7×7.

The third layer starts with a residual block comprising output channels 16 times the initial image channels, summing up to 1024 with a stride of size 2, resulting in a 14×14 output matrix. Thus, fed to an attention block with an encoder depth of 1, it results in the output size of the attention blocks to bottleneck 7×7. The final residual blocks comprise three residual blocks, each with 32 times the initial image channel as output channels, that is, 2048, and the first layer with stride size 2, resulting in a 7×7 matrix size. The final output layer of this module contains a pool size that encompasses the first and second indices of the presented result, by consuming the averaging 2-D pooling with the mentioned pool size and stride =1×1 and then flattened function and dropout activation, fleeting it to the output node dense with 7 output nodes (for 7 classes), kernel regularizer, and softmax activation function. The network configuration of the ResNet-92 and ResNet-56 attention modules is presented in Table 2.

#### 4.1.3. Attention EmoResNet

The third module in the LARNet is a customized residual attention model with ResNet acting as the backbone. The input shape taken in this module is 32×32, with 32 initial image channels. The first layer is comprised of a 2D Conv filter with size 32×32, kernel size 5×5, and padding = 1 applied to the batch normalization function, and ReLu activation function and 2D max-pooling with a pool size of 2×2, resulting in a 16×16 output matrix. The second layer consists of a residual and an attention block. The residual block has 32 input channels and 128 output channels, and the attention block has an encoder depth of 2. The third layer comprises single residual and attention blocks, with 128 input channels and 256 output channels with stride size of 2 in the residual block, resulting in an output size of 8×8 output size followed by the attention block with an encoder depth of 1. The fourth layer includes 256 input channels with 512 output channels and a stride size of 2, resulting in 4×4 output size followed by an attention block with an encoder depth of 1. The following are the final residual blocks comprised of three layers with the first one consisting of 512 input and 1024 output channels, and the remaining two consist of 1024 input and output channels each. This is followed by 2-D average pooling with pool size =4×4 and stride =1×1, resulting in an output size of 1×1. Finally, this is followed by the flatten function and output node with a dense function with 7 nodes and the softmax activation function.

### 4.2. Results and Analysis

#### 4.2.1. Implementation

The CASME II dataset [7,8] is a benchmark in facial micro expressions, recordings with a high temporal resolution of 200 fps, and relatively higher face resolution of 280 × 340 pixels. The dataset was collected at various time intervals, depending on the situation that suited the emotion. A separate set for cropped faces was provided. It was mainly used for testing because of its high resolution and properly labeled dataset, CASME II, provides confidence in the analysis of the model. Overall, 5000 images were trained on micro expressions using seven classes: ‘disgust’, ‘fear’, ‘happiness’, ‘others’, ‘repression’, ‘sadness’, and ‘surprise’. The most commonly used state-of-the-art ResNet network [18] was as a baseline method. The image was padded by four pixels on each side filled with 1 value on the 224 × 224 image patches. Data augmentation was computed, such as horizontal and vertical flip with a per-pixel RGB mean value, which was subtracted further. This work complies with the feedforward weight initialization mechanism for training the residual attention units using Nesterov SGD with a batch size of 64. A weight decay of 0.0001 with a momentum of 0.9 was initially set alongside an initial learning rate of 0.01. The overall network consisted of three stages with an equal number of residual attention models stacked at every stage. The weighted layers count in the trunk branch was given as 31m+9 (‘*m*’ represents the number of attention modules belonging to all individual stages). The training was terminated after 12,000 iterations.

#### 4.2.2. Residual Attention Learning

This experiment concludes the effectiveness of residual attention learning mechanisms on facial micro expressions. The size of the trained model was 224 MB with 21 M learnable parameters. As the notion of residual attention, learning is new in the field of facial emotions, more specifically, targeting micro-scale expressions, previous methods, such as naive residual networks or naive attention modules, are not suitable for detecting micro-scale facial expressions. The number of attention modules in every stage varies by m={1,2,3}.

**Figure 3:**
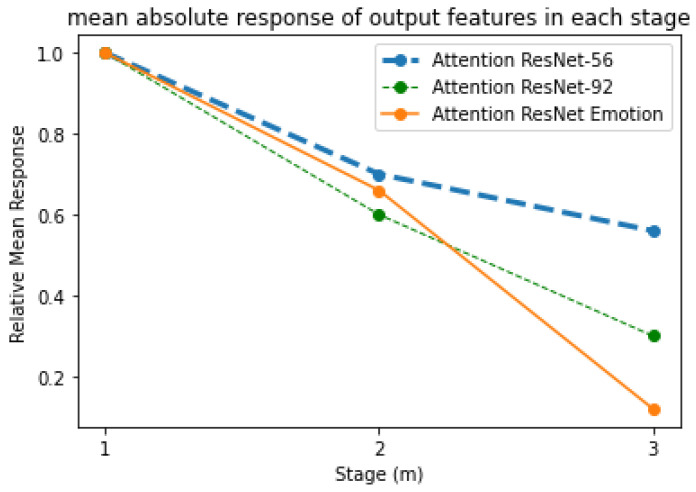
The mean absolute response rate from each stage on output features.

For a better understanding and analysis of residual attention modules, the authors calculated the mean absolute response of each attention stage present in the three modules. As shown in Figure 3, the performance and error rate decreased for each stage and attention network. The naive attention modules, in contrast, suffered obvious degradation with an increased number of attention modules. By contrast, LARNet performed with three residual networks with an increase in attention blocks, keeping the error rate performance decreasing manner as the stage keeps increasing. The attention modules, and 4 blocks in each module, are designed to suppress noise while keeping maximum information, aiming to avoid any information loss, whether useful or useless, by applying a dot product. However, it is known that the extracted information degrades severely by a dot product. Signal attenuation can be relieved by the residual attention learning using identical mapping that increase feature contrast in the attention blocks. These benefits are gained in terms of reduction in noise with no significant loss in information from the images that makes the optimization process a lot better while enhancing the represented features’ discrimination.

**Figure 4:**
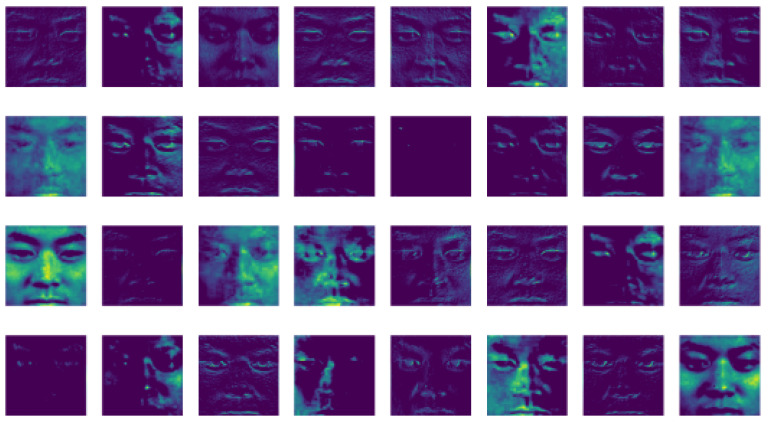
Attention ResNet-92 (Stage-1) hidden layer visualization of residual learning.

**Figure 5:**
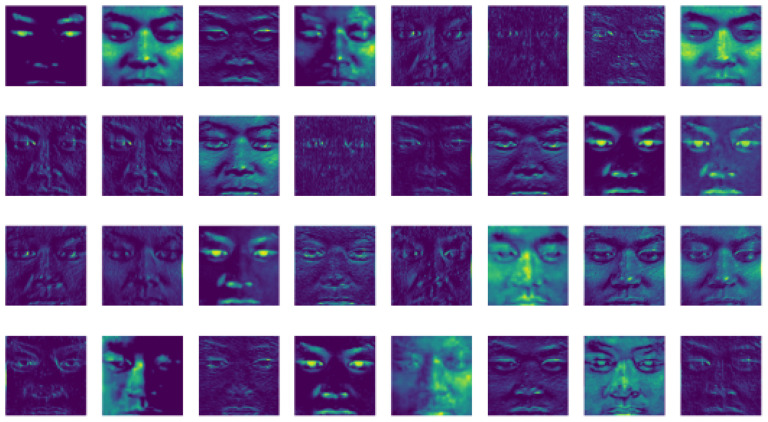
Attention ResNet-56 (Stage-2) hidden layer visualization of residual learning.

**Figure 6:**
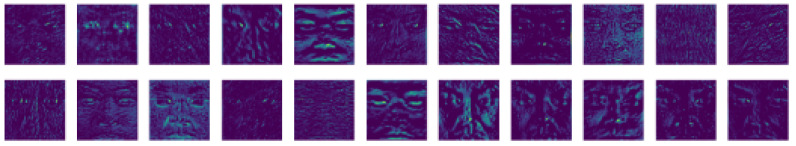
Attention ResNet Emotion (Stage-3) hidden layer visualization of residual learning.

Figure 4, Figure 5 and Figure 6 display the visualization of the hidden layer at each attention stage. This defines the actual computation of how information is extracted from the input images at each stage.

**Figure 7:**
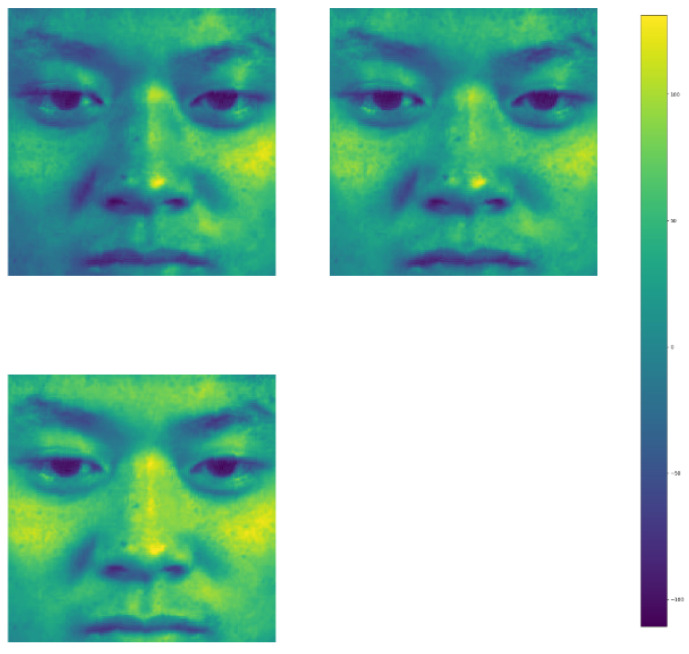
A close look at feature learning from a sample.

The results shown in Figure 3, contribute significantly to the encoder-decoders and local convolutions present in the attention modules. Attention ResNet-56 is used to construct Attention-Encoder-Decoder-56 and Attention-Local-Conv-56, and it is applied for the remaining two sequence networks. The presence of a soft attention optimization process in the attention modules benefits the multi-scale information by decreasing the error rate by proceeding with the other two residual attention modules. Figure 4, Figure 5, Figure 6 and Figure 7 represent residual learning in terms of the visualization of hidden layers of each stage. As each stage increments, micro-scale features are extracted in depth. At stage 3, the feature reaches the saturation point, at which point no feature can be extracted as shown in Figure 6, (in which most of the slots are empty and blank). A closer look at the feature extraction is shown in Figure 7 with possible locations of feature extraction visualized using heat maps.

#### 4.2.3. Performance Metrics

**Figure 8:**
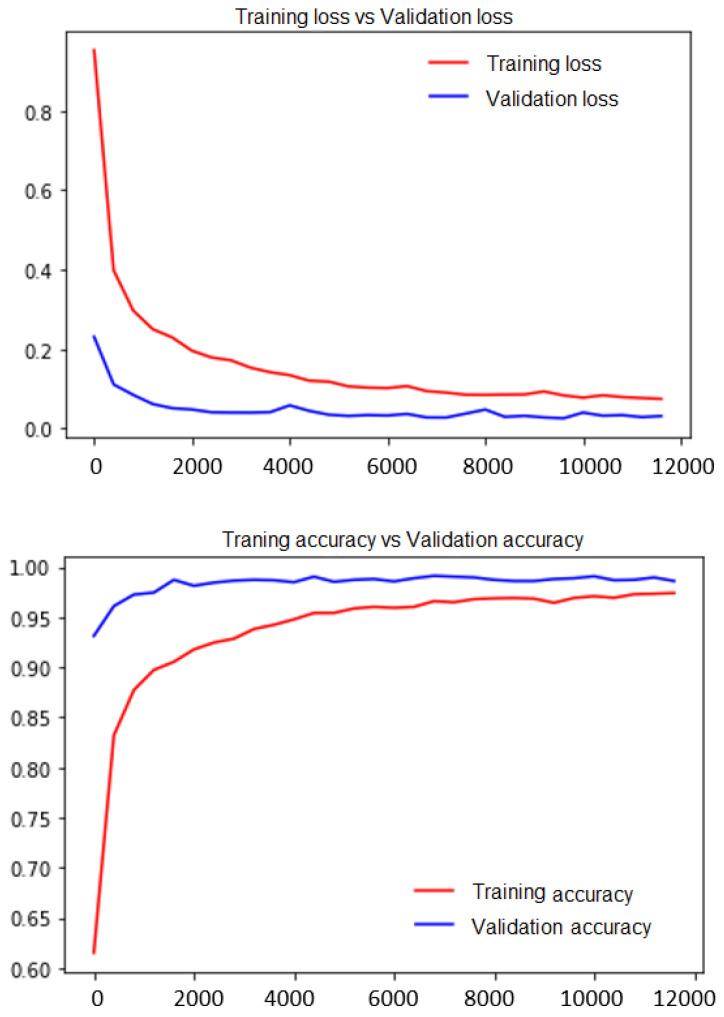
Computational performance on training dataset.

Figure 8 shows the training accuracy and loss performed on USF-HD, SMIC, and partly on CASME II. Most CASME II datasets are used as testing/prediction datasets. With over 5000 images of 7 classes, the training is performed on an Nvidia GTX 1080x Ti GPU, keeping the batch size to 64 to avoid memory allocation errors and is terminated at 12,000 steps. The training period time was more than 1 day for reaching a training accuracy of 97.41% with a loss of 0.0741, and the corresponding validation accuracy was noted at 98.61% with a loss of 0.309. The image dataset can be captured with a camera of at least 1080p and 200 fps as hardware configuration. Having less than 720p may change the results and may even provide false predictions as the micro expressions have to be captured on a very high-quality picture, so the model can dig deeper-based on the captured pixels. The authors evaluated the results of the data collected in three resolutions, that is, 1080p, 720p, and 480p, and below. As micro expressions are spontaneous and require a high-resolution, zoomed picture of the subject, 1080p and higher resolutions were subjected to above 87% for all emotions stated. Data captured at 720p exhibited a variation between 70% and 80%. False positives and false negatives were observed for data captured with a 720p resolution. Data captured with 480p and below showed low prediction levels and were deemed not suitable for evaluation. The video captured with 200 fps at 1080p resolution, that is, 1920 × 1080 pixel resolution was converted into 200 frames captured per second.

F1 score was plotted to evaluate the performance metrics on the training patch and testing patch [45,46]. Each feature and its weight are updated at each epoch. The F1 score is thus used for calculating the success rate of precision and recall. The precision and recall are the ratio of actual matches and correct predictions compared with total ground truths, respectively. Although, both of them are not sufficient for measuring the performance of the network. Therefore, the evaluation of the network is done with the F1 score, which is calculated using precision and recall as dependent parameters. F1 score is given by the parameters true positives (TP) as correct predictions, false negatives (FN) as false non-detections, and false positives (FP) as false correct predictions [45,46]. The mathematical computations for the above-mentioned parameters are as follows:

(7) $$Precision=\frac{TP}{TP+FP},$$

(8) $$Recall=\frac{TP}{TP+FN},$$

(9) $$F1\;\mathrm{score}=2\times \frac{Precision\times Recall}{Precision+Recall}.$$

**Figure 9:**
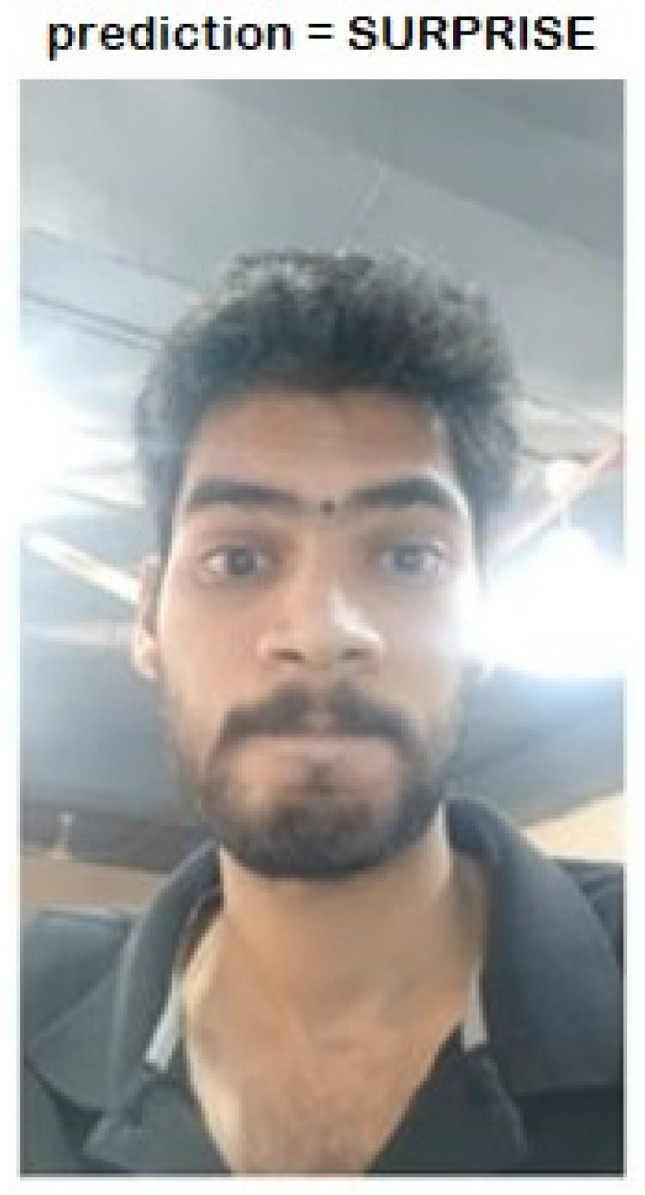
LARNet test results on real-time data.

The image classification model using the residual attention module, which was trained on over 5000 images of 7 classes, is not computationally complex. Hence, the model can be deployed on medium-scale edge devices for standalone testing. The inference speed tested on the GPU system is approximately 10 ms and on the CPU is approximately 60 s. The hardware configuration for testing the model was a full HD camera with 1920×1080 pixel resolution with 200 fps speed, placed remarkably close to the subject’s face. The results are tested in a GPU with 12 GB RAM, NVIDIA GTX 1080 Ti GPU. The model loading took approximately 180 s because it has a complex architecture and is heavy. However, after loading the model, the prediction time for each image is approximately 10 ms. The resultant captured data are a video of 200 fps, and these frames are then used for the prediction. Data captured with 720p lacked proper attention on the pixel values on the face, hence, a resolution of 1080p is recommended for prediction. The accuracy percentages of the data captured with different camera resolutions are listed in Table 3. The results vary even if the data were captured without a face detection model. The authors have used 128 landmarks to detect the face [47] and cropped the face when the data were captured with zoom out settings. However, it is better for the face to be very close to the camera lens because micro expressions are visible on a micro-level in nature and the closer the face is to the camera lens, the more accurate are the results. Figure 9 represents the LARNet model prediction of real-time data captured with a 1080p resolution camera. The frame in Figure 9 is the 76th frame and the total frames extracted for this result were approximately 600, and the video duration was 3 s.

**Figure 10:**
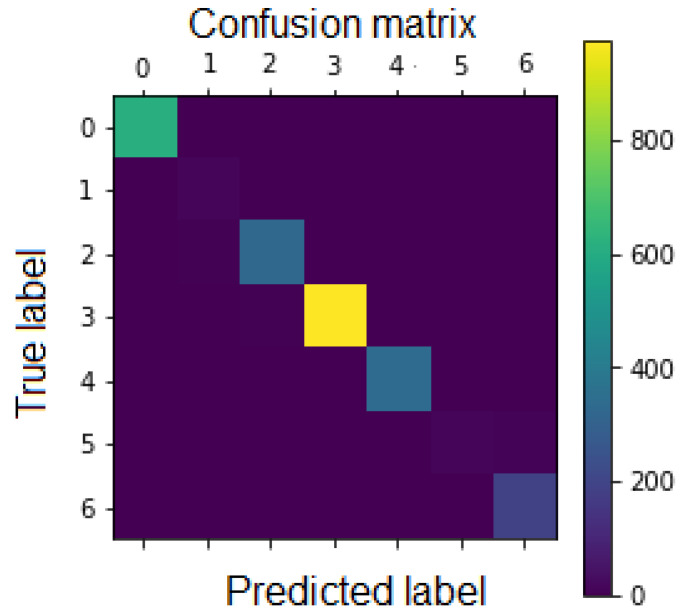
Confusion matrix on testing patch.

F1 score is plotted as listed in Table 4, which outlines LARNet performance of all 7 classes of CASME II dataset. The ‘Happiness’ and ‘Surprise’ classes outperformed the other classes. The other classes contain all mixed reactions, hence, accuracy is assured, as the other classes had a constant dataset, which leads to pledges high accuracy levels. Figure 10 visualizes the confusion matrix on the testing patch.

**Figure 11:**
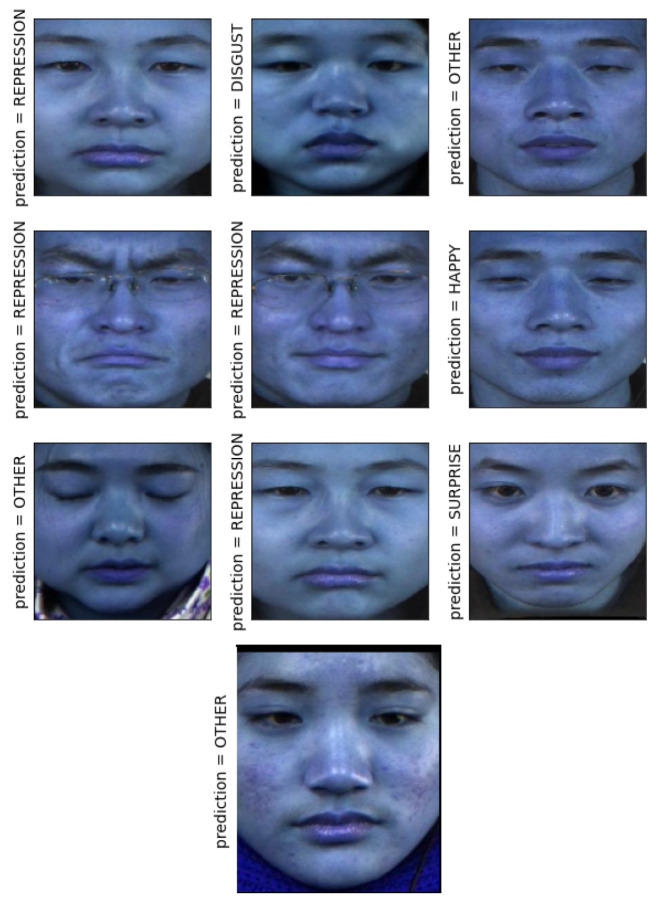
LARNet prediction on CASME II dataset (cropped faces).

**Figure 12:**
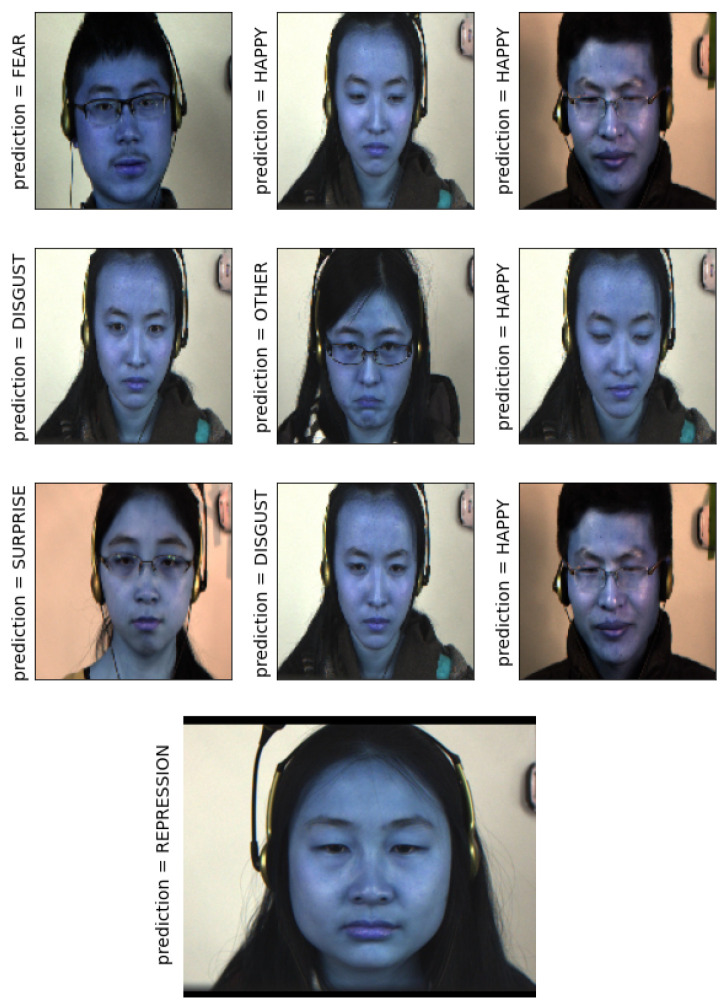
LARNet prediction on CASME II dataset (uncropped faces).

In Figure 11, the LARNet prediction results of the CASME II dataset are plotted. This figure presumes cropped images of the face. Figure 12 presumes the uncropped face images. The Cropped face images have resulted in ≈95% accuracy, whereas uncropped face images resulted in ≈92% accuracy.

#### 4.2.4. Comparison and Analysis with State-of-the-Art (SOTA) Models

Table 5 the proposed LARNet with other SOTA methods. Reddy et al. [48] involved extracting spatiotemporal information from faces and computing it with a 3D CNN network. MicroExpSTCNN involves extracting information from all available pixels, whereas MicroExpFuseNet involves extracting information from eyes and mouth regions. Although [48] has shown high accuracy levels using the CASME II and SMIC datasets, in real-time prediction, there are possibilities of false positives and false negatives due to computing with 3D CNN filters. Zhao et al. [49] proposed a local binary pattern—three orthogonal planes (LBP-TOP) method, wherein their model extracts features into the SVM classifier. Although their results are better than the hand-crafted methods, they fail to maintain high accuracy levels in real-time. Huang et al. [50] proposed local quantized patterns obtained from spatiotemporal information. This method learns dynamic patterns but did not show good results. Takalkar et al. [51] proposed data augmentation techniques for generating synthetic images and used those images for training with the CNN network. They gained to maintain important motion features to classify optical flow features of facial micro expressions. The main drawback of this method is that it may lose important temporal information by generating synthetic data. Li et al. [52] proposed a 3D flow for a CNN model for video-based micro expression recognition. It exhibits the same drawbacks as that of Reference [48]. The proposed LARNet surpasses all SOTA methods and performs slightly better than the 3D model in Reddy et al. [48]. Table 5 shows an overall accuracy comparison of LARNet with SOTA methods. A 2D landmark feature map (LFM) and CLFM [35] extracts the facial landmarks frame-wise and are fed to the CNN-LSTM hybrid architecture. This method focusses on all areas of the faces and hence the results are degraded instead of identifying the main focus parts in the video. Hence, the results are not fully accurate and works well by recognizing very few emotions. Lateral Accretive Hybrid Network (LEARNet) [53] uses a domain specific region with depth maps and compute with convolutional and ResNet layers. This is similar to our work, but our proposed LARNet has shown to be more accurate than LEARNet.

## 5. Conclusions

The proposed LARNet exhibits high accuracy levels for 6-specific classes, namely ‘happiness’, ‘fear’, ‘sadness’, ‘repression’, ‘disgust’, and ‘surprise’. These are the most important emotions much needed for any commercial usage and research analysis. The LARNet needed three stages to extract the fraction of a second which contained the crucial information on micro expressions that are needed for micro-scale feature extraction. Stage 1 mainly extracted macro-scale features. Stage 2 dug deeper inside the image frames but could not extract crucial parts, which can be a deciding factor. Hence, stage 3 was a customized network, which was included so that it could directly start from a micro-level feature extraction point, which reduces the training and eases feature learning by almost three times. Hence, the first two stages were taken from existing ResNet attention modules, which resulted in the extraction of features until the micro-scale starting point, followed by Stage 3 has taken forward, which just focused on micro-scale features. Hence, the network was designed according to “in divide and rule” to ease computation and accelerate learning by reducing the feature load on a single stage, rather by dividing it into three stages. This is how feature learning and training were built in a novel way.

The technical conclusion of the proposed LARNet architecture is it detects facial micro-expressions with certain hardware setup limitations. Although the results obtained in the real-time are very high and accurate, the face captured must be very high quality, and the distance between the camera and a user should not be more than 10 m. In real-time data capturing, results obtained with keeping camera more than 10 m away are mostly inaccurate. The same applies to computing side-angle faces. The face should be straight in order to obtain accurate results.

Detecting facial micro expressions is essential, especially in commercial malls. The proposed solution would be more feasible if the implementation is used for scene understanding when the user is looking at any product. Scene understanding in all commercial complexes are used for betterment of all users in the society to understand their needs and improve the products according to their feelings towards them. For achieving this, the user’s face must be captured in straight alignment in order to obtain accurate results for better analytics.

However, limitations of LARNet were encountered, which are discussed in the next section.

## 6. Limitations and Future Scope

The main limitation of this model is that the input image must be taken with at least a 200 fps camera and the high-resolution quality images must be provided. Images and videos are taken from normal standard cameras will fail to attain accurate results as the crucial micro-scale features are the deciding factor, and images or videos with noise often fails to get extracted at the micro-scale level. Another major limitation is that the model is trained for just six specific classes and that the seventh class is the ‘other’ category. There are more than 20 emotions. Hence, the unavailability of proper large datasets led to having to train with just 6 specific and 1 general class. As the model is built with multiple attention and residual modules, it is computationally complex; hence, medium-scale edge devices are suitable for running it remotely.

Future research is planned on visual-based lie detectors using micro expressions. As telling a lie results in an abrupt and minute fraction of facial emotion, a dataset can be collected, and research can progress in this area. To be able to deploy this model remotely, as necessary for commercial uses, the model can be cut down computationally to make it compatible with small-scale edge devices.

## Figures and Tables

**Table 1:** Experimental results on CASME2 micro expressions dataset implemented on proposed Lossless Attention Residual Network (LARNet) consisting of Attention ResNet-92, Attention ResNet-56 and custom build Attention ResNetEmotion blocks.

Activation Function	Attention Function	Error Rate
m(f)	Mixed Attention	3.13
c(f)	Channel Attention	5.6
s(f)	Spatial Attention	4.56

**Table 2:** Network configuration of Attention-56 and Attention-92 modules.

Layer	Output Size	Attention-56	Attention-92
Conv 1	112×112	7×7, 64, stride 2	
Max pooling	56×56	3×3, stride 2	
Residual unit	56×56	$\left(\begin{array}{cc} 1\times 1 & 64\\ 3\times 3 & 64\\ 1\times 1 & 256 \end{array}\right)\times 1$	
Attention module	56×56	Attention ×1	Attention ×1
Residual unit	28×28	$\left(\begin{array}{cc} 1\times 1 & 128\\ 3\times 3 & 128\\ 1\times 1 & 512 \end{array}\right)\times 1$	
Attention module	28×28	Attention ×1	Attention ×2
Residual unit	14×14	$\left(\begin{array}{cc} 1\times 1 & 256\\ 3\times 3 & 256\\ 1\times 1 & 1024 \end{array}\right)\times 1$	
Attention module	14×14	Attention ×1	Attention ×3
Residual unit	7×7	$\left(\begin{array}{cc} 1\times 1 & 512\\ 3\times 3 & 512\\ 1\times 1 & 2048 \end{array}\right)\times 3$	
Average pooling	1×1	7×7, stride 1	
FC, Softmax	1000		
Params $\times \;10^{6}$		31.9	51.3
FLOPS $\times \;10^{9}$		6.2	10.4
Trunk depth		56	92

**Table 3:** Results of real-time data of different camera resolutions for testing evaluation.

Camera Resolution	Accuracy Differentiation
1080p	>87%
720p	71–83%
<480p	<60%

**Table 4:** F1 score of LARNet performance on call 7 classes.

Expression Class	Precision	Recall	F1 Score
Happiness	0.97	0.95	0.95
Repression	0.92	0.90	0.90
Fear	0.85	0.89	0.86
Disgust	0.90	0.89	0.89
Surprise	0.97	0.95	0.95
Sadness	0.93	0.92	0.92
Others	0.80	0.75	0.77

**Table 5:** LARNet compared with state-of-the-art methods trained on CAS(ME)2 dataset.

Method	Results and Comparison with SOTA
Conventional CNN Classification [51]	78.02%
STCLQP [50]	64.02%
LBP-TOP [49]	42.72%
3D-FCNN [52]	55.49%
MicroExpSTCNN [48]	87.8%
Intermediate MicroExpFuseNet [48]	83.2%
Late MicroExpFuseNet [48]	79.3%
LFM-based (68×68 LFM) [35]	73.98%
CLFM-based (21×21 LFM) [35]	71.54%
LEARNet [53]	76.33%
**Proposed LARNet**	**91%**

## Data Availability

Data available in a publicly accessible repository: http://fu.psych.ac.cn/CASME/casme2-en.php.

## Abbreviations

The following abbreviations are used in this manuscript:

CNN	Convolutional Neural Network
DCNN	Deep Convolutional Neural Network
FN	False Negative
FP	False Positive
GPU	Graphics Processing Unit
LBP	Local Binary Pattern
LBP-MOP	Local Binary Pattern with Mean Orthogonal Planes
LBP-SIP	Linear Binary Pattern with Six Intersection Points
LBP-TOP	Local Binary Patterns on Three orthogonal Planes
LSTM	Long Short-Term Memory
NDP	Normalized Pixel Difference
SVM	Support Vector Machine
TP	True Positive
VGG	Visual Geometry Group
